# The association of Tai Chi exercise with the methylation levels of the IL20 promoter

**DOI:** 10.3389/fspor.2025.1585153

**Published:** 2026-01-02

**Authors:** Chiung-Hung Chiang, Oswald Ndi Nfor, Li-Yuan Chen, Min-Chen Wu, Yen-Chung Chen, Cheng-Feng Jan, Wen-Yu Lu, Yung-Po Liaw

**Affiliations:** 1 Department of Public Health and Institute of Public Health, Chung Shan Medical University, Taichung, Taiwan; 2 Department of Physical Therapy, Chung Shan Medical University, Taichung, Taiwan; 3 Office of Physical Education, Chung Yuan Christian University, Taoyuan, Taiwan; 4 Department of Neurology, China Medical University Hospital, Taichung, Taiwan; 5 Department of Neurology, Taichung Municipal Geriatric Rehabilitation General Hospital Managed by China Medical University, China Medical University, Taichung, Taiwan; 6 Department of Medical Imaging, Chung Shan Medical University Hospital, Taichung, Taiwan

**Keywords:** IL-20, Tai Chi exercise, methylation, gender, age

## Abstract

**Objective:**

This study aimed to investigate the associations between DNA methylation levels of the IL-20 and Tai Chi exercise.

**Methods and results:**

Data from the Taiwan Biobank, including 2,286 individuals aged 30–70, were analyzed. Methylation levels were assessed using the Infinium® MethylationEPIC BeadChipEPIC array. Statistical analyses were conducted to determine the associations between exercise types and methylation levels. The analysis revealed that participants who engaged in Tai Chi exhibited significantly higher methylation levels of the IL-20 promoter (mean *β* = 0.9405, SE ± 0.0019) compared to those who did not exercise (mean *β* = 0.9376, SE ± 0.0004). In univariate regression, Tai Chi exercise was positively associated with IL-20 promoter methylation (*β* = 0.00422, 95% CI: 0.00001–0.00843, *p* = 0.0493), whereas other forms of exercise showed a negative association (*β* = −0.00160, 95% CI: −0.00266 to −0.00053). Men exhibited lower IL-20 methylation levels, while older age and obesity showed similar trends. In the multivariate regression analysis, Tai Chi exercise remained positively associated with IL-20 promoter methylation (*β* = 0.00454, 95% CI: 0.00012–0.00896). Conversely, other exercise types were associated with a *β*-coefficient of −0.00125 (95% CI: −0.00239 to −0.00012).

**Conclusion:**

The findings suggest that Tai Chi exercise was associated with higher methylation levels of the IL-20 promoter. This association may indicate that the health benefits of Tai Chi are linked to immunological processes mediated by IL-20.

## Introduction

Tai Chi, an ancient Chinese martial art renowned for its slow, deliberate movements, meditation, and deep breathing, has increasingly captured attention for its diverse health benefits (1, 2). Research by Solianik et al. highlights Tai Chi as a versatile intervention that can be effectively adapted to pandemic conditions, significantly enhancing psychoemotional well-being, cognitive function, and motor learning among older adults (3). Likewise, Yeh's investigation revealed that a 12-week regimen of Tai Chi Chuan (TCC) can lead to improvements in functional mobility, health expectations, and regulatory T cell function (4). Further reinforcing its physical advantages, Siu and colleagues demonstrated that Tai Chi yields results comparable to traditional exercise, notably reducing waist circumference in middle-aged and older adults suffering from central obesity (5). Additionally, other studies indicate that the positive health effects of Tai Chi are largely independent of disease activity, suggesting that standardized health assessments could clarify the underlying mechanisms of these benefits (6). Wang's research also supports the safety and potential advantages of Tai Chi for individuals with functional class I or II Rheumatoid Arthritis (7).

In recent years, scholarly attention has shifted toward understanding the molecular mechanisms that underpin these health benefits, particularly through the lens of epigenetics (1). Epigenetics examines heritable changes in gene expression that occur without alterations to the DNA sequence itself, providing a compelling framework for exploring how lifestyle interventions like Tai Chi can positively influence health. Among various epigenetic modifications, DNA methylation plays a pivotal role in regulating gene expression and has been linked to a range of age-related diseases and inflammatory conditions (8). Emerging evidence suggests that regular Tai Chi practice may be associated with beneficial epigenetic changes, including significant alterations in DNA methylation patterns (1, 9). Notably, research by Ren et al. (2012) found that long-term practitioners of Tai Chi exhibited a reduced rate of age-related DNA methylation loss at several critical CpG sites, indicating that Tai Chi may contribute to delaying the effects of epigenetic aging (1).

IL-20, a cytokine with powerful inflammatory, angiogenic, chemoattractive, and osteoclastogenic properties, plays a critical role in various stages of rheumatoid arthritis (RA) progression, as well as in obesity-related inflammation and insulin resistance (10–12). Dysregulation of IL-20 and its receptors is frequently observed in non-small cell lung cancer (NSCLC) through epigenetic mechanisms, with the silencing of IL-20RA correlating with poorer disease-free survival outcomes in NSCLC patients (13). Given the potential of exercise, including Tai Chi, to modulate inflammatory responses (14), and considering the central role of IL-20 in inflammation (15), investigating the methylation status of the IL-20 promoter region could yield valuable insights into the molecular pathways linking Tai Chi practice to enhanced immune function and overall health.

Recognizing the interplay between the benefits of Tai Chi exercise and the role of IL-20 in immunology, our study sought to explore IL-20 methylation as a potential optimization strategy. To our knowledge, no previous research has examined this specific relationship. Thus, we investigate the effects of Tai Chi exercise on the methylation levels of the IL-20 promoter, while factoring in the influences of gender, age, and obesity. Through this exploration, we aim to deepen the understanding of how Tai Chi may exert its beneficial effects via epigenetic modifications.

## Materials and methods

This study aimed to investigate the association between different types of Tai Chi exercise and DNA methylation levels, specifically the mean methylation of the IL20 promoter. The study population comprised individuals aged 30–70 years, all of Taiwanese nationality, without foreign ancestry or prior history of cancer. Data were obtained from 2,286 participants with available methylation measurements in the Taiwan Biobank. Before data collection, all participants provided written informed consent. Participants were required to hold Taiwanese nationality and self-report no foreign ancestry to ensure population genetic homogeneity and reduce confounding from ethnic methylation differences. This criterion follows Taiwan Biobank's standard recruitment policy and IRB-approved protocol. All data were de-identified prior to release by the Taiwan Biobank. The study used coded datasets under IRB approval (CS1-20009), ensuring participant anonymity and compliance with ethical regulations.

DNA methylation was assessed using DNA extracted from blood samples and analyzed with the Infinium® MethylationEPIC BeadChipEPIC array (Illumina Inc.). Methylation data were normalized using the Illumina® GenomeStudio V2011.1 software (16). To account for blood cell-type heterogeneity, correction was performed in R using the Reference-Free Adjustment for Cell-Type composition (ReFACTor) method (17). Demographic variables, including age, sex, body mass index (BMI), body fat percentage, lifestyle factors (such as smoking and alcohol consumption), genetic information, and blood cell-type composition, were retrieved from the database.

### Definitions of variables

Exercise types were categorized as follows: “No exercise” referred to individuals who did not engage in regular exercise; “Other exercise” denoted regular exercise excluding Tai Chi, Wai-Tan Gong, Nei-Tan Gong, Falun Gong, Yuanji Dance, and Xiang Gong, which share meditative, slow-movement, low-intensity, and mind–body coordination features. Participants were asked via questionnaire whether they had engaged in regular exercise over the past three months. Regular exercise was defined as participation in physical activity at least three times per week, with each session lasting more than 30 min. For smoking status, “Never smokers” were defined as those who had never smoked or had quit smoking for more than six months, while “Former or current smokers” referred to individuals who had smoked for more than six months and either quit or continued to smoke at the time of the survey. For alcohol consumption, “Never drinkers” were defined as individuals who never drank alcohol or consumed less than 150 cc per week, whereas “Former or current drinkers” referred to those who had quit drinking for at least six months or consumed at least 150 cc of alcohol per week consistently for six months or longer.

### Data analysis

Data analysis was performed using SAS 9.4 software (SAS Institute, Cary, NC). Statistical analyses aimed to examine the association between exercise types and IL20 promoter mean methylation levels. Chi-square tests were used to compare the distribution of categorical variables across exercise types, while analysis of variance (ANOVA) was employed to compare the mean values among participants with different exercise types. Categorical variables were presented as numbers (percentages), and continuous variables were expressed as means ± standard error (mean ± SE). Univariate regression models were used to assess the associations between methylation levels [IL20 promoter mean, i.e., the mean *β* value of CpG sites annotated to the TSS200 region (≤200 bp upstream of the transcription start site) of IL-20] and each variable. Multivariate linear regression models were then applied to evaluate the relationship between exercise types and methylation levels (IL20 promoter mean). Our statistical model also adjusted for 5 PCA components, including leucocyte types.

To test for multicollinearity among independent variables, the variance inflation factor (VIF) was calculated. Generally, a VIF value below 10 indicates no serious multicollinearity, whereas a VIF value above 10 suggests substantial correlation among predictors, which may lead to unstable coefficient estimates.

## Results

The study examined the characteristics of a population divided into three groups: those who did not exercise (*n* = 1,340), those who engaged in other forms of exercise (*n* = 913), and those who practiced Tai Chi (*n* = 33), as detailed in Table 1. Notably, the average *β* values of the IL20 promoter, which reflect methylation levels, varied significantly among these groups. The Tai Chi group exhibited the highest average *β* value (0.9405, SE ± 0.0019), suggesting a greater level of methylation compared to the no exercise group (0.9376, SE ± 0.0004). The gender distribution also varied significantly among the groups (*p* < 0.0001). In the no-exercise group, 53.51% were women, while the other exercise group had a lower proportion of women at 44.14%. The Tai Chi group had a gender distribution of 51.52% women and 48.48% men, indicating a relatively balanced representation. Regarding the body composition, the Tai Chi group had a notably lower body fat percentage (24.78%) compared to the other exercise group (26.40%) and the no exercise group (28.10%). Among participants in the No Exercise group, 843 individuals (62.91%) were under 50 years of age, while 497 individuals (37.09%) were aged between 50 and 70 years. In the Other Exercise group, 273 individuals (29.90%) were under 50 years, and 640 individuals (70.10%) were aged 50–70 years. Conversely, the Tai Chi Exercise group included only 3 individuals (9.09%) under 50 years, with the majority, 30 individuals (90.91%), aged between 50 and 70 years.

**Table 1:** Basic characteristics of the study population.

Variables	No exercise	Other exercise	Tai-Chi exercise	*p*-value
	(*n* = 1,340)	(*n* = 913)	(*n* = 33)	
IL20 promoter (Mean *β*±SE)	0.9376 ± 0.0004	0.9350 ± 0.0005	0.9405 ± 0.0019	<0.0001
Gender, *n* (%)				<0.0001
Women	717 (53.51)	403 (44.14)	17 (51.52)	
Men	623 (46.49)	510 (55.86)	16 (48.48)	
Age (years)				<0.0001
<50 years	843 (62.91)	273 (29.90)	3 (9.09)	
50 ≤ Age ≤ 70	497 (37.09)	640 (70.10)	30 (90.91)	
Body fat percentage	28.10 ± 0.21	26.40 ± 0.24	24.78 ± 1.50	<0.0001
BMI (Kg/m^2^)				<0.0001
Underweight	47 (3.51)	12 (1.31)	1 (3.03)	
Normal	615 (45.90)	454 (49.73)	20 (60.61)	
Overweight	356 (26.57)	286 (31.33)	8 (24.24)	
Obese	322 (24.03)	161 (17.63)	4 (12.12)	
Smoking status				0.4290
Never smoked	1,003 (74.85)	669 (73.27)	27 (81.82)	
Former or current smoker	337 (25.15)	244 (26.73)	6 (18.18)	
Alcohol consumption				0.3660
Never	1,217 (90.82)	815 (89.27)	31 (93.94)	
Former or current consumer	123 (9.18)	98 (10.73)	2 (6.06)	

BMI, body mass index, *β*, beta coefficient, SE, standard error.

The analysis of various factors affecting IL20 promoter methylation was further explored through univariate models, as outlined in Table 2. In this analysis, Tai Chi exercise was positively associated with IL20 promoter methylation, as indicated by a *β*-coefficient of 0.00422 (95% CI: 0.00001–0.00843; *p* = 0.0493), whereas other exercise groups were negatively associated (*β* = −0.00160, 95% CI: −0.00266 to −0.00053). Gender differences were observed, with men showing a negative association (*β* = −0.00235, 95% CI: −0.00338 to 0.00133) compared to women. Both age and current smoking were also associated with negative impacts on IL20 promoter methylation, with those aged 50 years and older (50 ≤ Age ≤ 70) showing a *β*-coefficient of −0.00151 (95% CI: −0.00266 to −0.00035), and current smokers showing a *β*-coefficient of −0.00254 (95% CI: −0.00370 to −0.00139). The analysis of alcohol consumption revealed no significant association.

**Table 2:** The impact of each factor on the IL20 promoter using univariate models.

Features	*β*-coefficient	95% CI		*p*-value
Exercise (ref: no exercise)				
Other exercise	−0.00160	−0.00266	−0.00053	0.0033
Tai-Chi exercise	0.00422	0.00001	0.00843	0.0493
Gender (ref: women)				
Men	−0.00235	−0.00338	−0.00133	<0.0001
Age (ref: <50 years)				
50 ≤ Age ≤ 70	−0.00151	−0.00266	−0.00035	0.0109
Body fat percentage	0.00008	0.00002	0.00015	0.0170
BMI (ref: Normal)				
Underweight	0.00146	−0.00179	0.00471	0.3795
Overweight	−0.00046	−0.00158	0.00066	0.4228
Obesity	−0.00055	−0.00179	0.00068	0.3805
Smoking (ref: Never)				
Former or current smoker	−0.00254	−0.00370	−0.00139	<0.0001
Alcohol consumption (ref: Never)				
Former or current consumer	−0.00126	−0.00295	0.00042	0.1414

The model was adjusted for 5 PCA components.

In the multiple regression analysis presented in Table 3, which accounted for various factors simultaneously, Tai Chi exercise remained positively associated with IL20 promoter methylation (0.00454 (95% CI: 0.00012–0.00896), reinforcing the earlier findings. Conversely, “other exercise” was associated with a *β*-coefficient of −0.00125 (95% CI: −0.00239 to −0.00012). The age group of 50 years and older (50 ≤ Age ≤ 70) had a *β*-coefficient of −0.00107 (95% CI: −0.00232 to 0.00018) and a *p*-value of 0.0926. Although this suggests a negative association with IL20 promoter methylation, it does not reach statistical significance at the conventional threshold. Other factors, such as body fat percentage and alcohol consumption, did not display significant associations in this model. In this study, all independent variables exhibited VIF values below 5, indicating low intercorrelation among predictors.

**Table 3:** The impact of each factor on the IL-20 promoter using a multiple linear regression model.

Features	*β*-coefficient	95% CI		*p*-value	VIF
Exercise (ref: no exercise)					
Other exercise	−0.00125	−0.00239	−0.00012	0.0305	1.15364
Tai-Chi exercise	0.00454	0.00012	0.00896	0.0440	1.03569
Gender (ref: women)					
Men	−0.00098	−0.00297	0.00102	0.3387	3.72294
Age (ref: <50 years)					
50 ≤ Age ≤ 70	−0.00107	−0.00232	0.00018	0.0926	1.45132
Body fat percentage	0.00001	−0.00012	0.00016	0.7962	4.24458
BMI (ref: Normal)					
Underweight	0.00089	−0.00266	0.00444	0.6221	1.20000
Overweight	−0.00013	−0.00163	0.00137	0.8659	1.70565
Obesity	−0.00036	−0.00249	0.00176	0.7368	2.82032
Smoking status (ref: Never smoked)					
Former or current smoker	−0.00238	−0.00373	−0.00103	0.0005	1.28936
Alcohol consumption (ref: Never)					
Former or current consumer	0.00036	−0.00148	0.00221	0.7000	1.11917

The model was adjusted for 5 PCA components (including leucocyte type).

If the Variance Inflation Factor (VIF) is less than 5, it indicates that multicollinearity is not a major concern and is generally considered to suggest little to no multicollinearity. Since the VIFs of the following variables are all below 5, there is no multicollinearity.

Table 4 shows the results of the sensitivity analysis across seven models, each excluding different variables before performing multivariate regression with the remaining factors. Specifically, Model 1 excluded alcohol consumption yielding a *β* value of 0.00453, *p* = 0.0443), Model 2 excluded smoking status (*β* = 0.00472, *p* = 0.0369), Model 3 excluded BMI (*β* = 0.00451, *p* = 0.0451), Model 4 excluded both alcohol consumption and smoking status (*β* = 0.00473, *p* = 0.0363), Model 5 excluded alcohol consumption and BMI (*β* = 0.00451, *p* = 0.0454), Model 6 excluded smoking status and BMI (*β* = 0.00469, *p* = 0.0378), and Model 7 excluded alcohol consumption, smoking status, and BMI (*β* = 0.00470, *p* = 0.0373). In all models, the main effect of Tai Chi exercise remained significantly positively associated with IL20 promoter methylation, demonstrating the robustness of this association regardless of which variables were excluded.

**Table 4:** Sensitivity analysis of covariate impact on IL-20 promoter methylation in Tai Chi practitioners.

Features	Model 1		Model 2		Model 3		Model 4		Model 5		Model 6		Model 7	
	*Β*	*p*-value	*Β*	*p*-value	*Β*	*p*-value	*Β*	*p*-value	*Β*	*p*-value	*Β*	*p*-value	*Β*	*p*-value
Exercise (ref: no exercise)														
Other exercise	−0.00125	0.0306	−0.00119	0.0404	−0.00127	0.0284	−0.00119	0.0405	−0.00127	0.0285	−0.00120	0.0376	−0.00120	0.0376
Tai-Chi exercise	0.00453	0.0443	0.00472	0.0369	0.00451	0.0451	0.00473	0.0363	0.00451	0.0454	0.00469	0.0378	0.00470	0.0373
Gender (ref: women)														
Men	−0.00094	0.3550	−0.00182	0.0664	−0.00128	0.0771	−0.00188	0.0557	−0.00124	0.0833	−0.00213	0.0018	−0.00219	0.0010
Age (ref: <50 years)														
50 ≤ Age ≤ 70	−0.00107	0.0929	−0.00101	0.1144	−0.00107	0.0934	−0.00101	0.1144	−0.00106	0.0937	−0.00100	0.1149	−0.00100	0.1149
Body fat rate	0.00002	0.7897	0.00001	0.8619	−0.00001	0.8939	0.00001	0.8696	−0.00001	0.9038	−0.00001	0.7783	−0.00001	0.7664
BMI (ref: Normal)														
Underweight	0.00090	0.6197	0.00009	0.6180	—	—	0.00090	0.6200	—	—	—	—	—	—
Overweight	−0.00012	0.8715	−0.00016	0.8380	—	—	−0.00016	0.8319	—	—	—	—	—	—
Obesity	−0.00037	0.7355	−0.00033	0.7371	—	—	−0.00036	0.7383	—	—	—	—	—	—
Smoking status (ref: Never smoked)														
Former or current smoker	−0.00232	0.0005	—	—	−0.00238	0.0005	—	—	0.00232	0.0005	—	—	—	—
Alcohol consumption (ref: Never)														
Former or current consumer	—	—	−0.00092	0.7171	0.00037	0.6954	—	—	—	—	−0.00033	0.7201	—	—

All the models adjusted for 5 PCA components.

Table 5 summarizes the sensitivity analysis of covariates, including socioeconomic factors and comorbidities, on IL-20 promoter methylation in Tai Chi practitioners. This analysis aimed to assess the robustness of the association between Tai Chi exercise and IL20 promoter methylation. Specifically, Model 1 excluded alcohol consumption, resulting in a *β* coefficient of 0.00452 (*p* = 0.0450). Model 2 excluded smoking status, yielding a *β* of 0.00481 (*p* = 0.0325). Model 3 excluded BMI, with a *β* of 0.00457 (*p* = 0.0425), Model 4 excluded both alcohol consumption and smoking status, producing a *β* of 0.00455 (*p* = 0.0437), Model 5 excluded alcohol consumption and BMI, demonstrating a *β* of 0.00479 (*p* = 0.0334), Model 6 excluded smoking status and BMI, resulting in a *β* of 0.00483 (*p* = 0.0319), and Model 7 excluded alcohol consumption, yielding a *β* of 0.00481 (*p* = 0.0327). In all models, Tai Chi exercise remained significantly positively associated with IL20 promoter methylation, demonstrating the robustness of this association regardless of which variables were excluded.

**Table 5:** Sensitivity analysis of covariates, including socioeconomic factors and comorbidities, on IL-20 promoter methylation in Tai Chi practitioners.

Features	Model 1		Model 2		Model 3		Model 4		Model 5		Model 6		Model 7	
	*Β*	*p*-value	*Β*	*p*-value	*Β*	*p*-value	*Β*	*p*-value	*Β*	*p*-value	*Β*	*p*-value	*Β*	*p*-value
Exercise (ref: no exercise)														
Other exercise	−0.00123	0.0343	−0.00133	0.0220	−0.00123	0.0336	−0.00123	0.0341	−0.00133	0.0221	−0.00133	0.0219	−0.00133	0.0221
Tai-Chi exercise	0.00452	0.0450	0.00481	0.0325	0.00457	0.0425	0.00455	0.0437	0.00479	0.0334	0.00483	0.0319	0.00481	0.0327
Gender (ref: women)														
Men	−0.00104	0.3086	−0.00099	0.3297	−0.00098	0.3399	−0.00101	0.3247	−0.00103	0.3114	−0.00096	0.3437	−0.00101	0.3237
Age (ref: <50 years)														
50 ≤ Age ≤ 70	−0.00096	0.1345	−0.00098	0.1246	−0.00103	0.1092	−0.00093	0.1488	−0.00087	0.1743	−0.00095	0.1355	−0.00085	0.1872
Body fat percentage	0.00003	0.7156	0.00002	0.7779	0.00002	0.7576	0.00003	0.7008	0.00003	0.7128	0.00002	0.7657	0.00003	0.7018
BMI (ref: Normal)														
Underweight	0.00091	0.6168	0.00079	0.6622	0.00092	0.6098	0.00092	0.6113	0.00078	0.6644	0.00080	0.6575	0.00079	0.6599
Overweight	−0.00016	0.8357	−0.00024	0.7536	−0.00017	0.8284	−0.00016	0.8308	−0.00024	0.7559	−0.00024	0.7501	−0.00024	0.7527
Obesity	−0.00036	0.7426	−0.00023	0.8338	−0.00028	0.7990	−0.00031	0.7789	−0.00025	0.8137	−0.00019	0.8625	−0.00022	0.8412
Smoking status (ref: Never smoked)														
Former or current smoker	−0.00233	0.0008	−0.00245	0.0004	−0.00234	0.0007	−0.00231	0.0009	−0.00241	0.0005	−0.00244	0.0004	−0.00240	0.0006
Alcohol consumption (ref: Never)														
Former or current consumer	0.00038	0.6886	0.00047	0.6175	0.00035	0.7122	0.00036	0.7003	0.00049	0.6041	0.00046	0.6270	0.00048	0.6129
Education level (ref: elementary and under)														
Junior and senior high school	0.00130	0.3582	—	—	—	—	0.00127	0.3701	0.00133	0.3453	—	—	0.00131	0.3542
University and above	0.00151	0.2822	—	—	—	—	0.00149	0.2889	0.00161	0.2500	—	—	0.00160	0.2549
Asthma (ref: No)														
Yes	—	—	−0.00557	0.0001	—	—	—	—	−0.00561	0.0001	−0.00555	0.0002	−0.00559	0.0001
Diabetes (ref: No)														
Yes	—	—	—	—	−0.00059	0.5598	−0.00057	0.5743	—	—	−0.00045	0.6588	−0.00043	0.6738

All the models were adjusted for 5 PCA components.

## Discussion

The results of this study indicate a significant positive association between Tai Chi exercise and IL-20 promoter methylation levels. The observed mean *β* value for IL-20 promoter methylation in the Tai Chi group was notably higher than that of the no-exercise group, reinforcing the potential immunological benefits of Tai Chi practice. The multivariate analysis further supports the robustness of this association, as Tai Chi remained positively correlated with IL-20 methylation even after controlling for factors. This finding aligns with existing literature that has highlighted positive associations of Tai Chi exercise and various health outcomes, including improved motor function, cognitive function, and reduction of falls in patients with neurodegenerative diseases (3, 4, 18). Additionally, Tai Chi has demonstrated beneficial effects comparable to standard physical therapy in treating knee osteoarthritis, and randomized controlled trials have consistently shown significant health benefits associated with both Tai Chi and Qigong (19, 20).

The potential positive impact of Tai-Chi exercise on the regulation of IL-20 is noteworthy, given that IL-20 is a multifunctional cytokine involved in inflammation and insulin resistance. These implications are particularly relevant for autoimmune diseases such as psoriasis and rheumatoid arthritis, underscoring the need for further research to elucidate the underlying mechanisms and clinical significance of IL-20 promoter methylation in Tai Chi practitioners (10). Understanding the intricate interplay of cytokines, such as IL-19, IL-20, and IL-24, with their receptors and their influence on autoimmunity can offer valuable insights into the therapeutic potential of Tai Chi exercise (21).

Tai-Chi exercise has been associated with various other health benefits, such as improving psychoemotional state, cognition, functional mobility, regulatory T cell function, and reducing waist circumference in individuals with central obesity (4, 5). Our study acknowledges that while the primary focus was on Tai Chi exercise and IL-20 promoter methylation, factors such as age, gender, and obesity may influence methylation patterns and health outcomes. Specifically, the results indicated that men exhibited a lower IL-20 methylation level (*β* = −0.00235) and individuals aged 50 years and older showed a similar negative trend (*β* = −0.00151). Additionally, obesity was associated with reduced IL-20 methylation, highlighting the need for further investigation into how these demographic factors interact with the epigenetic effects of Tai Chi practice.

While our study provides valuable insights, it is important to acknowledge several limitations. One key limitation is the lack of data on medication use and dietary habits, both of which may significantly influence methylation patterns independently of Tai Chi exercise. Although we accounted for several covariates, the absence of information on these factors restricts our ability to fully assess their potential confounding effects. Additionally, gene expression was not directly measured. However, we focused our methylation analysis on CpG sites annotated to the TSS200 region (within 200 bp upstream of the transcription start site), a well-recognized core promoter region strongly associated with transcriptional regulation. Methylation changes at TSS200 are known to exert the greatest influence on gene expression, as they can directly interfere with transcription factor binding and RNA polymerase II recruitment. Prior analyses have shown that methylation within TSS200 exhibits the strongest inverse correlation with mRNA expression across the genome (22, 23). Even though mRNA and protein levels were not assessed in the present study, our focus on TSS200 methylation provides biologically meaningful insights into the transcriptional potential of the target gene. That is, IL-20 promoter methylation may down-regulate gene expression, thereby potentially attenuating IL-20–mediated pro-inflammatory signaling. Thus, higher methylation observed in Tai-Chi exercisers could reflect reduced inflammatory tone, consistent with previous reports linking mind–body exercise to lower systemic inflammation. The lower IL-20 methylation observed in the “other exercise” group suggests that exercise modality may influence epigenetic regulation differently. Mind–body exercises such as Tai Chi may preferentially modulate stress- or inflammation-related epigenetic pathways, whereas high-intensity physical activities might induce distinct molecular responses.

Another limitation of the study is the uncertainty surrounding the clinical significance of the observed increase in IL-20 promoter methylation (approximately 0.4%). While existing literature suggests that even subtle changes in DNA methylation in regulatory regions can lead to meaningful transcriptional modulation, the direct implications for immune function and disease outcomes remain unclear. Consequently, the findings provide preliminary epidemiological evidence but lack clinical validation. Future longitudinal and interventional studies will be essential to evaluate the translational relevance of IL-20 methylation changes in relation to health improvements and immune phenotypes.

It is also important to acknowledge that the cross-sectional nature of this study limits our ability to draw causal inferences regarding the relationship between Tai Chi practice and IL-20 methylation. While our findings suggest an association, we cannot determine whether engagement in Tai Chi leads to changes in methylation levels or if individuals with certain epigenetic profiles are more inclined to participate in such activities. Furthermore, the relatively small size of the Tai Chi group (33 participants out of 2,286) undermines the statistical power of our analyses and may have introduced uncertainty into the subgroup findings.

These limitations underscore the need for future research employing longitudinal methodologies and larger sample sizes to clarify the directionality of these relationships and enhance the generalizability of the results. By addressing these gaps, subsequent studies can provide a clearer understanding of the potential impact of Tai Chi practice on epigenetic modifications.

## Conclusions

In conclusion, the observed higher methylation level of the IL-20 promoter in Tai Chi practitioners suggests a potential association between Tai Chi exercise and immunological processes mediated by IL-20. The findings align with existing literature that highlights the health benefits of Tai Chi, including its positive effects on physical and mental well-being. However, the clinical implications of these results require further investigation. Future research should focus on longitudinal and interventional studies to elucidate the causal relationships between Tai Chi practice, IL-20 promoter methylation, and associated health outcomes.

## Data Availability

The data supporting the findings of this study are available from the Taiwan Biobank data source; however, restrictions apply to the availability of these data, which were used under license for the current research and are therefore not publicly available. Data are, however, available from the authors upon reasonable request and with permission of Taiwan Biobank.
